# Solving synaptic PET‐biomarker puzzle in Alzheimer's and other tauopathies with UCB‐J

**DOI:** 10.1002/alz.090648

**Published:** 2025-01-09

**Authors:** Miriam Scarpa, Elisavet Vallera, Sira Ausellé‐Bosch, Filipa Monteiro Rocha, Buse Esra Mercan, Agneta K Nordberg, Amit Kumar

**Affiliations:** ^1^ Karolinska Institutet, Stockholm Sweden; ^2^ University of Crete, Heraklion Greece; ^3^ University of Girona, Girona Spain; ^4^ Hacettepe University, Ankara Turkey; ^5^ Theme Inflammation and Aging, Karolinska University Hospital, Stockholm Sweden

## Abstract

**Background:**

Synaptic loss is an eminent feature of tauopathies. The recently developed novel SV2A PET‐tracer UCB‐J has shown great promise in tracking synaptic loss in tauopathies. However, there have been discrepancies between the in vivo findings and a lack of mechanistic insight. Many of these studies indicated potential correlations between tau deposition, atrophy, cognitive‐impairment, and loss of UCB‐J binding. Hence, we found it of utmost relevance to perform extensive pre‐clinical validation of UCB‐J in tauopathic brains to gain a deeper understanding of synaptic loss for future PET‐biomarker validation.

**Method:**

We applied an innovative multidimensional approach of postmortem brain imaging techniques (large and small frozen brain section autoradiography) and radioligand binding studies (saturation, competition, and regional distribution) alongside in‐depth biochemical analyses using a large repertoire of synaptic markers in Alzheimer’s disease (AD), progressive supranuclear palsy (PSP), and control brains.

**Result:**

Saturation/competition studies showed one‐binding site (Kd: 3‐10 nM) for UCB‐J in the frontal cortex and globus pallidus (GP) of AD, PSP, and control brains. UCB‐J regional binding studies at the synaptosomal level clearly showed synaptic loss in different regions of AD and PSP brains as compared to control brains. In AD brains, the loss of UCB‐J binding showed the following order: Hippocampus > Frontal cortex > Temporal Cortex > Parietal Cortex > Cerebellum. Immunoblot analyses showed a positive‐correlation between UCB‐J binding and expression of synaptic proteins SV2A, synaptophysin, and synaptotagmin and demonstrated association with GABA/glutaminergic neurotransmitter machinery in GP of PSP brains. Interestingly, cortical and sub‐cortical 4R‐tau load distinctly affected synaptic marker protein levels in PSP brains. Large brain‐section autoradiography studies in AD brains indicated potential ‘off‐target’ interaction of UCB‐J with nuclear P‐tau. PSP brain's small brain‐section autoradiography also showed higher binding in cortical regions, specifically, in the parietal cortex where significantly (*p=0.034) higher ^3^H‐UCB‐J binding was observed compared to CN.

**Conclusion:**

Our studies explicitly showed the high specificity of UCB‐J for SV2A and demonstrated synaptic deficits/loss in AD and PSP brain regions. Ongoing studies will shed more light on the interplay between different synaptic and pathological markers and help establish the reliability of UCB‐J as a definite marker of synaptic density/loss for tauopathies.

